# The Nooscope manifested: AI as instrument of knowledge extractivism

**DOI:** 10.1007/s00146-020-01097-6

**Published:** 2020-11-21

**Authors:** Matteo Pasquinelli, Vladan Joler

**Affiliations:** 1 Media Philosophy Department, Karlsruhe University of Arts and Design, Karlsruhe, Germany; 2 New Media Department, Academy of Arts, University of Novi Sad, Novi Sad, Serbia

**Keywords:** Nooscope, Political economy, Mechanised knowledge, Information compression, Ethical machine learning

## Abstract

Some enlightenment regarding the project to mechanise reason. The assembly line of machine learning: data, algorithm, model. The training dataset: the social origins of machine intelligence. The history of AI as the automation of perception. The learning algorithm: compressing the world into a statistical model. All models are wrong, but some are useful. World to vector: the society of classification and prediction bots. Faults of a statistical instrument: the undetection of the new. Adversarial intelligence vs. statistical intelligence: labour in the age of AI.

## Some enlightenment regarding the project to mechanise reason

The Nooscope is a cartography of the limits of artificial intelligence, intended as a provocation to both computer science and the humanities. Any map is a partial perspective, a way to provoke debate. Similarly, this map is a manifesto—of AI dissidents. Its main purpose is to challenge the mystifications of artificial intelligence. First, as a technical definition of *intelligence* and, second, as a political form that would be *autonomous* from society and the human.^1^ In the expression ‘artificial intelligence’, the adjective ‘artificial’ carries the myth of the technology’s autonomy; it hints to caricatural ‘alien minds’ that self-reproduce in silico but, actually, mystifies two processes of proper alienation; the growing geopolitical autonomy of hi-tech companies and the invisibilization of workers’ autonomy worldwide. The modern project to mechanise human reason has clearly mutated, in the twenty first century, into a corporate regime of knowledge extractivism and epistemic colonialism.^2^ This is unsurprising, since machine learning algorithms are the most powerful algorithms for information compression.

The purpose of the Nooscope map is to secularize AI from the ideological status of ‘intelligent machine’ to one of knowledge instruments. Rather than evoking legends of alien cognition, it is more reasonable to consider machine learning as an *instrument of knowledge magnification* that helps to perceive features, patterns, and correlations through vast spaces of data beyond human reach. In the history of science and technology, this is no news; it has already been pursued by optical instruments throughout the histories of astronomy and medicine.^3^ In the tradition of science, machine learning is just a *Nooscope*, an instrument to see and navigate the space of knowledge (from the Greek *skopein* ‘to examine, look’ and *noos* ‘knowledge’).

Borrowing the idea from Gottfried Wilhelm Leibniz, the Nooscope diagram applies the analogy of optical media to the structure of all machine learning apparatuses. Discussing the power of his *calculus ratiocinator* and ‘characteristic numbers’ (the idea to design a numerical universal language to codify and solve all the problems of human reasoning), Leibniz made an analogy with instruments of visual magnification such as the microscope and telescope. He wrote: ‘Once the characteristic numbers are established for most concepts, mankind will then possess a new instrument which will enhance the capabilities of the mind to a far greater extent than optical instruments strengthen the eyes, and will supersede the microscope and telescope to the same extent that reason is superior to eyesight’ (Leibniz 1677, p. 23). Although the purpose of this text is not to reiterate the opposition between quantitative and qualitative cultures, Leibniz’s credo need not be followed. Controversies cannot be conclusively computed. Machine learning is not the ultimate form of intelligence.

Instruments of measurement and perception always come with inbuilt aberrations. In the same way that the lenses of microscopes and telescopes are never perfectly curvilinear and smooth, the *logical lenses* of machine learning embody faults and biases. To understand machine learning and register its impact on society is to study the degree by which social data are diffracted and distorted by these lenses. This is generally known as the debate on bias in AI, but the political implications of the logical form of machine learning are deeper. Machine learning is not bringing a new dark age but one of diffracted rationality, in which, as it will be shown, an episteme of causation is replaced by one of automated correlations. More in general, AI is a new regime of truth, scientific proof, social normativity and rationality, which often does take the shape of a *statistical hallucination*. This diagram manifesto is another way to say that AI, the king of computation (patriarchal fantasy of mechanised knowledge, ‘master algorithm’ and *alpha machine*) is naked. Here, we are peeping into its black box.
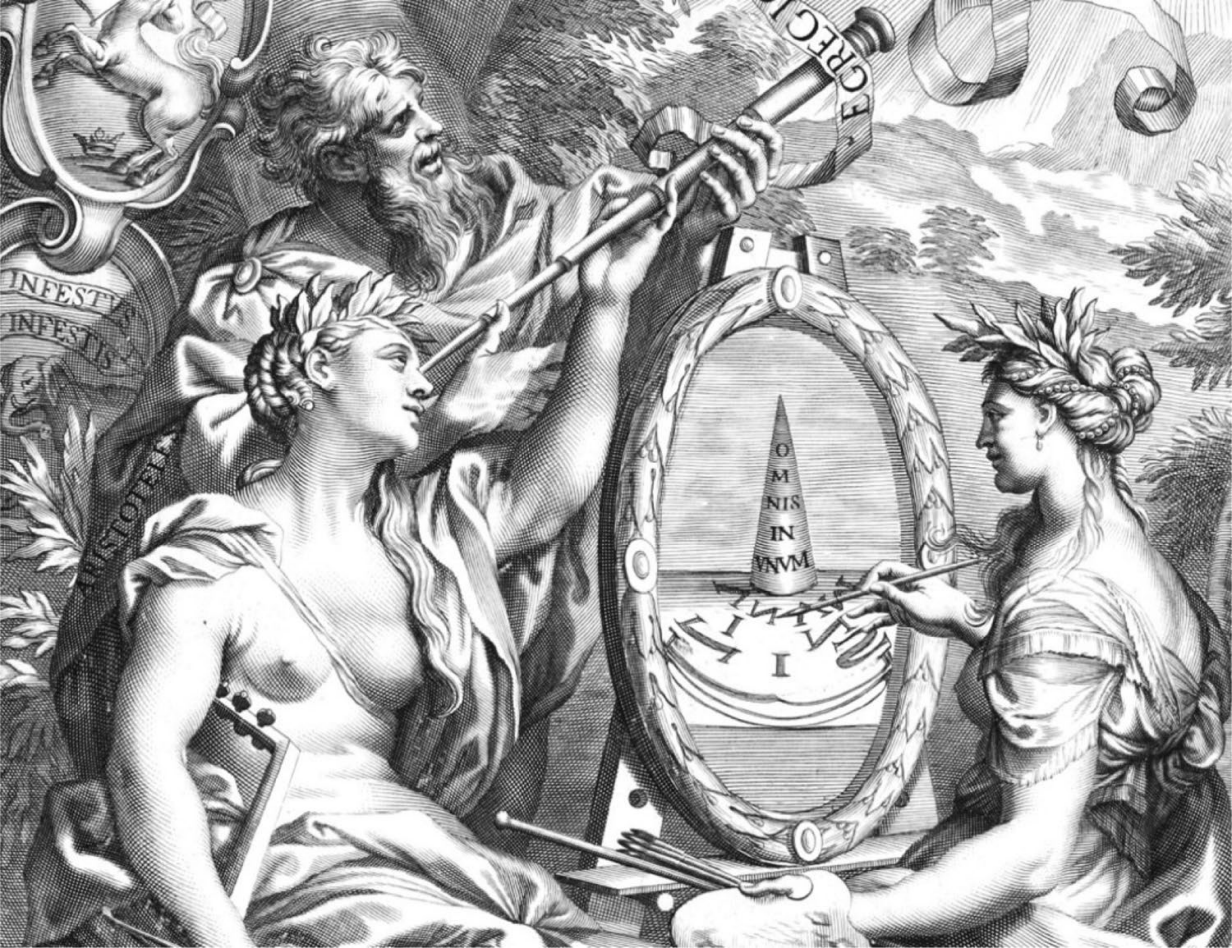


On the invention of metaphors as instrument of knowledge magnification.

Emanuele Tesauro, *Il canocchiale aristotelico* [The Aristotelian Telescope], frontispiece of the 1670 edition, Turin.

## The assembly line of machine learning: data, algorithm, model

The history of AI is a history of experiments, machine failures, academic controversies, epic rivalries around military funding, popularly known as ‘winters of AI.’^4^ Although corporate AI today describes its power with the language of ‘black magic’ and ‘superhuman cognition’, current techniques are still at the experimental stage (Campolo and Crawford 2020). AI is now at the same stage as when the steam engine was invented, before the laws of thermodynamics necessary to explain and control its inner workings, had been discovered. Similarly, today, there are efficient neural networks for image recognition, but there is no *theory of learning* to explain why they work so well and how they fail so badly. Like any invention, the paradigm of machine learning consolidated slowly, in this case through the last half-century. A master algorithm has not appeared overnight. Rather, there has been a gradual construction of a method of computation that still has to find a common language. Manuals of machine learning for students, for instance, do not yet share a common terminology. How to sketch, then, a critical grammar of machine learning that may be concise and accessible, without playing into the paranoid game of defining General Intelligence?

As an instrument of knowledge, machine learning is composed of an object to be observed (*training dataset*), an instrument of observation (*learning algorithm*) and a final representation (*statistical model*). The assemblage of these three elements is proposed here as a spurious and baroque diagram of machine learning, extravagantly termed Nooscope.^5^ Staying with the analogy of optical media, the information flow of machine learning is like a light beam that is projected by the training data, compressed by the algorithm and diffracted towards the world by the lens of the statistical model.

The Nooscope diagram aims to illustrate two sides of machine learning at the same time: *how it works and how it fails*—enumerating its main components, as well as the broad spectrum of errors, limitations, approximations, biases, faults, fallacies and vulnerabilities that are native to its paradigm.^6^ This double operation stresses that AI is not a monolithic paradigm of rationality but a spurious architecture made of adapting techniques and tricks. Besides, the limits of AI are not simply technical but are imbricated with human bias. In the Nooscope diagram, the essential components of machine learning are represented at the centre, *human biases* and interventions on the left, and *technical biases* and limitations on the right. Optical lenses symbolize biases and approximations representing the compression and distortion of the information flow. The total bias of machine learning is represented by the central lens of the statistical model through which the perception of the world is diffracted.

The limitations of AI are generally perceived today thanks to the discourse on bias—the amplification of gender, race, ability, and class discrimination by algorithms. In machine learning, it is necessary to distinguish between historical bias, dataset bias, and algorithm bias, all of which occur at different stages of the information flow.^7^*Historical bias* (or world bias) is already apparent in society before technological intervention. Nonetheless, the naturalisation of such bias, that is the silent integration of inequality into an apparently neutral technology is by itself harmful (Eubanks 2018).^8^ Paraphrasing Michelle Alexander, Ruha Benjamin has called it the New Jim Code: ‘the employment of new technologies that reflect and reproduce existing inequalities but that are promoted and perceived as more objective or progressive than the discriminatory systems of a previous era’ (Benjamin 2019, p. 5). *Dataset bias* is introduced through the preparation of training data by human operators. The most delicate part of the process is data labelling, in which old and conservative taxonomies can cause a distorted view of the world, misrepresenting social diversities and exacerbating social hierarchies (see below the case of ImageNet).

*Algorithmic bias* (also known as machine bias, statistical bias or model bias, to which the Nooscope diagram gives particular attention) is the further amplification of historical bias and dataset bias by machine learning algorithms. The problem of bias has mostly originated from the fact that machine learning algorithms are among the most efficient for *information compression*, which engenders issues of information resolution, diffraction and loss.^9^ Since ancient times, algorithms have been procedures of an economic nature, designed to achieve a result in the shortest number of steps consuming the least amount of resources: space, time, energy and labour (Pasquinelli (forthcoming) The eye of the master. Verso, London). The arms race of AI companies is, still today, concerned with finding the simplest and fastest algorithms with which to capitalise data. If information compression produces the maximum rate of profit in corporate AI, from the societal point of view, it produces discrimination and the loss of cultural diversity.

While the social consequences of AI are popularly understood under the issue of bias, the common understanding of technical limitations is known as the *black box* problem. The black box effect is an actual issue of deep neural networks (which filter information so much that their chain of reasoning cannot be reversed) but has become a generic pretext for the opinion that AI systems are not just inscrutable and opaque, but even ‘alien’ and out of control.^10^ The black box effect is part of the nature of any experimental machine at the early stage of development (it has already been noticed that the functioning of the steam engine remained a mystery for some time, even after having been successfully tested). The actual problem is the black box rhetoric, which is closely tied to conspiracy theory sentiments in which AI is an occult power that cannot be studied, known, or politically controlled.

## The training dataset: the social origins of machine intelligence

Mass digitalisation, which expanded with the Internet in the 1990s and escalated with datacentres in the 2000s, has made available vast resources of data that, for the first time in history, are free and unregulated. A regime of *knowledge extractivism* (then known as Big Data) gradually employed efficient algorithms to extract ‘intelligence’ from these open sources of data, mainly for the purpose of predicting consumer behaviours and selling ads. The knowledge economy morphed into a novel form of capitalism, called *cognitive capitalism* and then *surveillance capitalism,* by different authors (Corsani et al. 2004; Zuboff 2019). It was the Internet information overflow, vast datacentres, faster microprocessors and algorithms for data compression that laid the groundwork for the rise of AI monopolies in the twenty first century.

What kind of cultural and technical object is the dataset that constitutes the source of AI? The quality of *training data* is the most important factor affecting the so-called ‘intelligence’ that machine learning algorithms extract. There is an important perspective to take into account, to understand AI as a Nooscope. Data are the first source of value and intelligence. Algorithms are second; they are the machines that compute such value and intelligence into a model. However, training data are never raw, independent and unbiased (they are already themselves ‘algorithmic’) (Gitelman 2013). The carving, formatting and editing of training datasets are a laborious and delicate undertaking, which is probably more significant for the final results than the technical parameters that control the learning algorithm. The act of selecting one data source rather than another is the profound mark of human intervention into the domain of the ‘artificial’ minds.
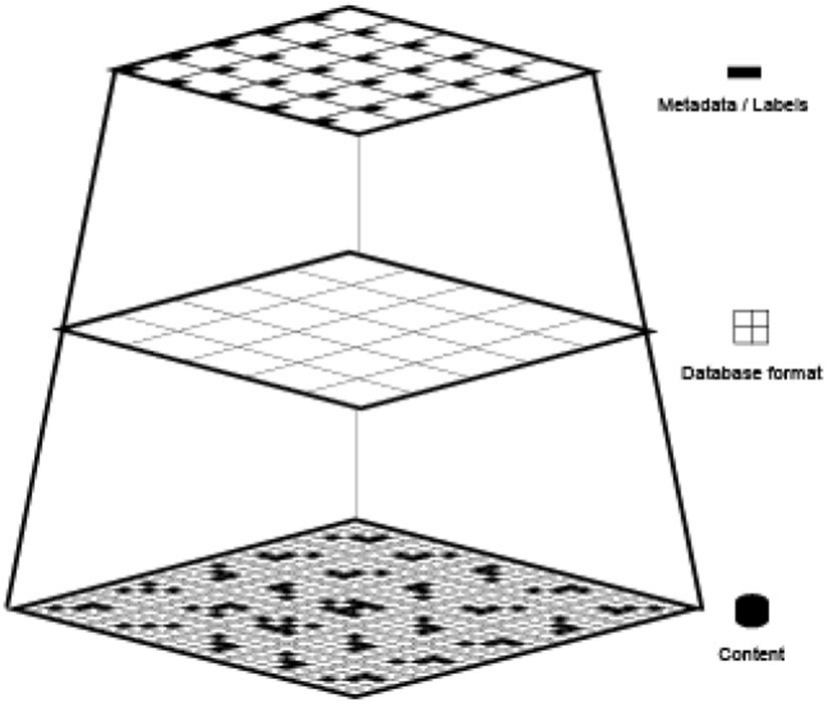


The training dataset is a *cultural construct*, not just a technical one. It usually comprises input data that are associated with ideal output data, such as pictures with their descriptions, also called labels or metadata.^11^ The canonical example would be a museum collection and its archive, in which artworks are organised by metadata such as author, year, medium, etc. The semiotic process of assigning a name or a category to a picture is never impartial; this action leaves another deep human imprint on the final result of machine cognition. A training dataset for machine learning is usually composed through the following steps: (1) production: labour or phenomena that produce information; (2) capture: encoding of information into a data format by an instrument; (3) formatting: organisation of data into a dataset; (4) labelling: in supervised learning, the classification of data into categories (metadata).

Machine intelligence is trained on vast datasets that are accumulated in ways neither technically neutral nor socially impartial. Raw data do not exist, as it is dependent on human labour, personal data, and social behaviours that accrue over long periods, through extended networks and controversial taxonomies.^12^ The main training datasets for machine learning (NMIST, ImageNet, Labelled Faces in the Wild, etc.) originated in corporations, universities, and military agencies of the Global North. But taking a more careful look, one discovers a profound division of labour that innervates into the Global South via crowdsourcing platforms that are used to edit and validate data.^13^ The parable of the *ImageNet* dataset exemplifies the troubles of many AI datasets. ImageNet is a training dataset for Deep Learning that has become the de facto benchmark for image recognition algorithms: indeed, the Deep Learning revolution started in 2012 when Alex Krizhevsky, Ilya Sutskever and Geoffrey Hinton won the annual ImageNet challenge with the convolutional neural network AlexNet.^14^ ImageNet was initiated by computer scientist Fei-Fei Li back in 2006.^15^ Fei-Fei Li had three intuitions to build a reliable dataset for image recognition. First, to download millions of free images from web services such as Flickr and Google. Second, to adopt the computational taxonomy *WordNet* for image labels.^16^ Third, to outsource the work of labelling millions of images via the crowdsourcing platform Amazon Mechanical Turk. At the end of the day (and of the assembly line), anonymous workers from all over the planet were paid few cents per task to label hundreds of pictures per minute according to the WordNet taxonomy: their labour resulted in the engineering of a controversial cultural construct. AI scholars Kate Crawford and artist Trevor Paglen have investigated and disclosed the sedimentation of racist and sexist categories in ImageNet taxonomy: see the legitimation of the category ‘failure, loser, nonstarter, unsuccessful person’ for a hundred arbitrary pictures of people (Crawford and Paglen 2019).

The voracious data extractivism of AI has caused an unforeseeable backlash on digital culture: in the early 2000s, Lawrence Lessig could not predict that the large repository of online images credited by *Creative Commons* licenses would a decade later become an unregulated resource for face recognition surveillance technologies. In similar ways, personal data are continually incorporated without transparency into privatised datasets for machine learning. In 2019 artist and AI researcher, Adam Harvey for the first time disclosed the nonconsensual use of personal photos in training datasets for face recognition. Harvey’s disclosure caused Stanford University, Duke University and Microsoft to withdraw their datasets amidst a major *privacy infringement scandal* (Harvey 2019; Murgia 2019). Online training datasets trigger issues of data sovereignty and civil rights that traditional institutions are slow to counteract (see the European General Data Protection Regulation).^17^ If 2012 was the year in which the Deep Learning revolution began, 2019 was the year in which its sources were discovered to be vulnerable and corrupted.
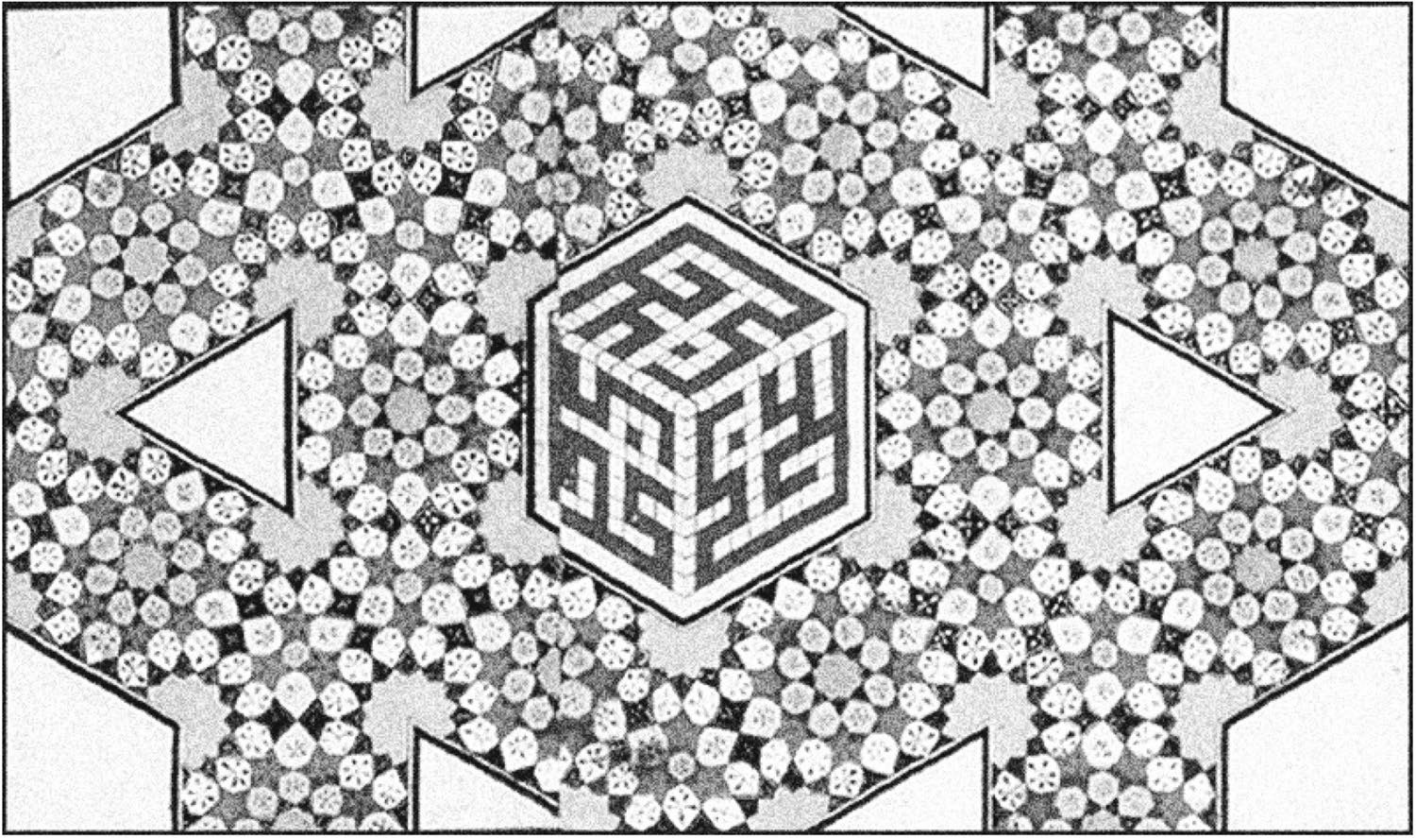


Combinatorial patterns and Kufic scripts, Topkapi scroll, ca. 1500, Iran.

## The history of AI as the automation of perception

The need to demystify AI (at least from the technical point of view) is understood in the corporate world too. Head of Facebook AI and godfather of convolutional neural networks Yann LeCun reiterates that current AI systems are not sophisticated versions of cognition, but rather, of perception. Similarly, the Nooscope diagram exposes the skeleton of the AI black box and shows that AI is not a thinking automaton but an algorithm that performs *pattern recognition*. The notion of pattern recognition contains issues that must be elaborated upon. What is a pattern, by the way? Is a pattern uniquely a visual entity? What does it mean to read social behaviours as patterns? Is pattern recognition an exhaustive definition of intelligence? Most likely not. To clarify these issues, it would be good to undertake a brief archaeology of AI.

The archetype machine for pattern recognition is Frank Rosenblatt’s *Perceptron*. Invented in 1957 at Cornell Aeronautical Laboratory in Buffalo, New York, its name is a shorthand for ‘Perceiving and Recognizing Automaton’ (Rosenblatt 1957). Given a visual matrix of 20 × 20 photoreceptors, the Perceptron can learn how to recognise simple letters. A visual pattern is recorded as an impression on a network of artificial neurons that are firing up in concert with the repetition of similar images and activating one single output neuron. The output neuron fires 1 = true, if a given image is recognised, or 0 = false, if a given image is not recognised.

The automation of perception, as a visual montage of pixels along a computational assembly line, was originally implicit McCulloch and Pitt’s concept of artificial neural networks (McCulloch and Pitts 1947). Once the algorithm for visual pattern recognition survived the ‘winter of AI’ and proved efficient in the late 2000s, it was applied also to non-visual datasets, properly inaugurating the age of Deep Learning (the application of pattern recognition techniques to all kinds of data, not just visual). Today, in the case of self-driving cars, the patterns that need to be recognised are objects in road scenarios. In the case of automatic translation, the patterns that need to be recognised are the most common sequences of words across bilingual texts. Regardless of their complexity, from the numerical perspective of machine learning, notions such as image, movement, form, style, and ethical decision can all be described as statistical distributions of pattern. In this sense, pattern recognition has truly become a new *cultural technique* that is used in various fields. For explanatory purposes, the Nooscope is described as a machine that operates on three modalities: *training*, *classification*, and *prediction*. In more intuitive terms, these modalities can be called: pattern extraction, pattern recognition, and pattern generation.

Rosenblatt’s Perceptron was the first algorithm that paved the way to machine learning in the contemporary sense. At a time when ‘computer science’ had not yet been adopted as definition, the field was called ‘computational geometry’ and specifically ‘connectionism’ by Rosenblatt himself. The business of these neural networks, however, was to calculate a statistical inference. What a neural network computes is not an exact pattern but the *statistical distribution of a pattern*. Just scraping the surface of the anthropomorphic marketing of AI, one finds another technical and cultural object that needs examination: the *statistical model*. What is the statistical model in machine learning? How is it calculated? What is the relationship between a statistical model and human cognition? These are crucial issues to clarify. In terms of the work of demystification that needs to be done (also to evaporate some naïve questions), it would be good to reformulate the trite question ‘Can a machine think?’ into the theoretically sounder questions ‘Can a statistical model think?’, ‘Can a statistical model develop consciousness?’, et cetera.

## The learning algorithm: compressing the world into a statistical model

The algorithms of AI are often evoked as alchemic formulas, capable of distilling ‘alien’ forms of intelligence. But what do the algorithms of machine learning really do? Few people, including the followers of artificial general intelligence (AGI), bother to ask this question. Algorithm is the name of a process, whereby a machine performs a calculation. The product of such machine processes is a statistical model (more accurately termed an ‘algorithmic statistical model’). In the developer community, the term ‘algorithm’ is increasingly replaced with ‘model.’ This terminological confusion arises from the fact that the statistical model does not exist separately from the algorithm: somehow, the statistical model exists inside the algorithm under the form of distributed memory across its parameters. For the same reason, it is essentially impossible to visualise an algorithmic statistical model, as is done with simple mathematical functions. Still, the challenge is worthwhile.

In machine learning, there are many *algorithm architectures*: simple Perceptron, deep neural network, Support Vector Machine, Bayesian network, Markov chain, autoencoder, Boltzmann machine, etc. Each of these architectures has a different history (often rooted in military agencies and corporations of the Global North). Artificial neural networks started as simple computing structures that evolved into complex ones which are now controlled by a few *hyperparameters* that express millions of *parameters*.^18^ For instance, convolutional neural networks are described by a limited set of hyperparameters (number of layers, number of neurons per layer, type of connection, behaviour of neurons, etc.) that project a complex topology of thousands of artificial neurons with millions of parameters in total. The algorithm starts as a blank slate and, during the process called training, or ‘learning from data', adjusts its parameters until it reaches a good representation of the input data. In image recognition, as already seen, the computation of millions of parameters has to resolve into a simple binary output: 1 = true, a given image is recognised; or 0 = false, a given image is not recognised.^19^
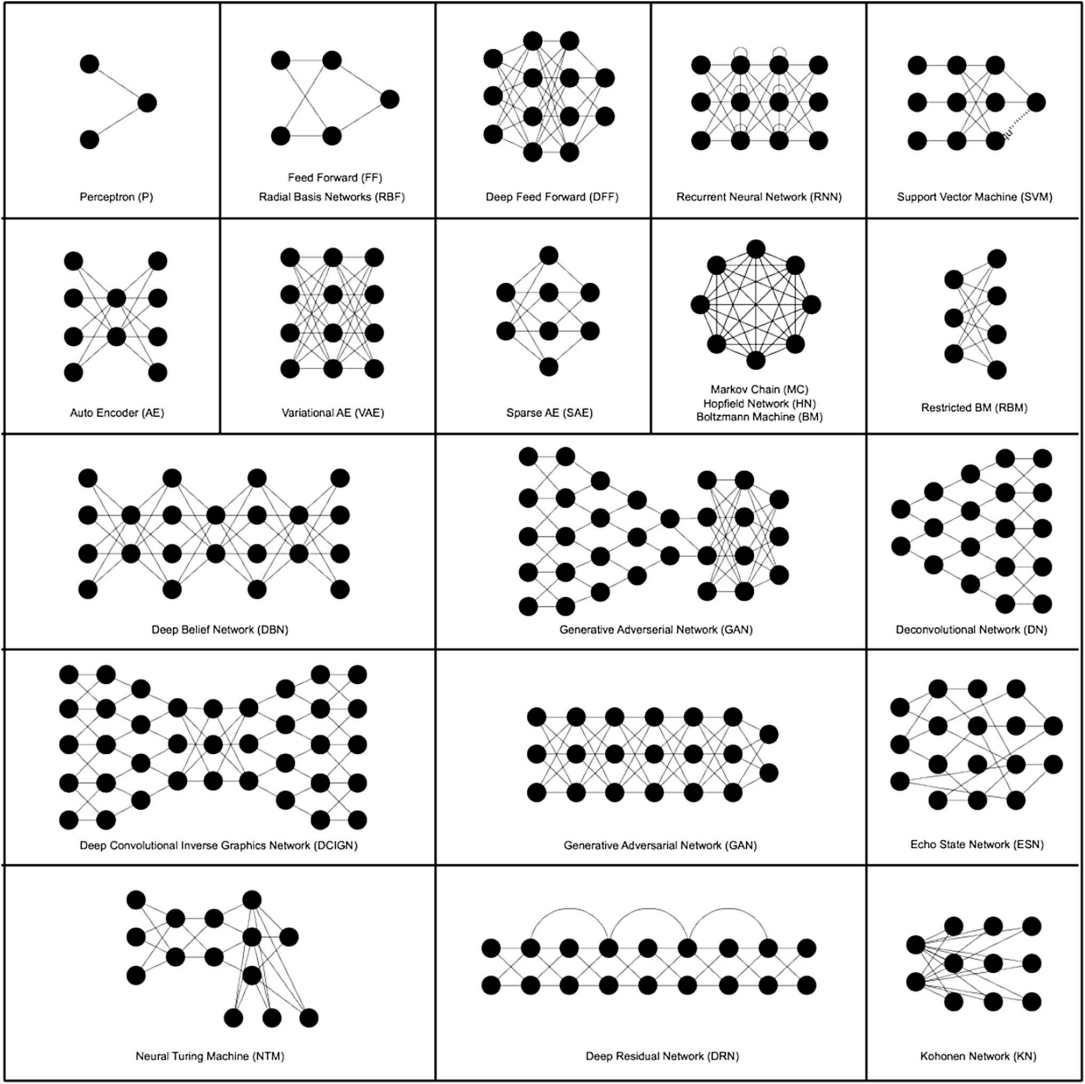


Source: https://www.asimovinstitute.org/neural-network-zoo

Attempting an accessible explanation of the relationship between algorithm and model, let us have a look at the complex Inception v3 algorithm, a deep convolutional neural network for image recognition designed at Google and trained on the ImageNet dataset. Inception v3 is said to have a 78% accuracy in identifying the label of a picture, but the performance of ‘machine intelligence’ in this case can be measured also by the proportion between the size of training data and the trained algorithm (or model). ImageNet contains 14 million images with associated labels that occupy approximately 150 gigabytes of memory. On the other hand, Inception v3, which is meant to represent the information contained in ImageNet, is only 92 megabytes. The ratio of compression between training data and model partially describes also the rate of information diffraction. A table from the Keras documentation compares these values (numbers of parameters, layer depth, file dimension and accuracy) for the main models of image recognition.^20^ This is a brutalist but effective way to show the relation between model and data, to show how the ‘intelligence’ of algorithms is measured and assessed in the developer community.
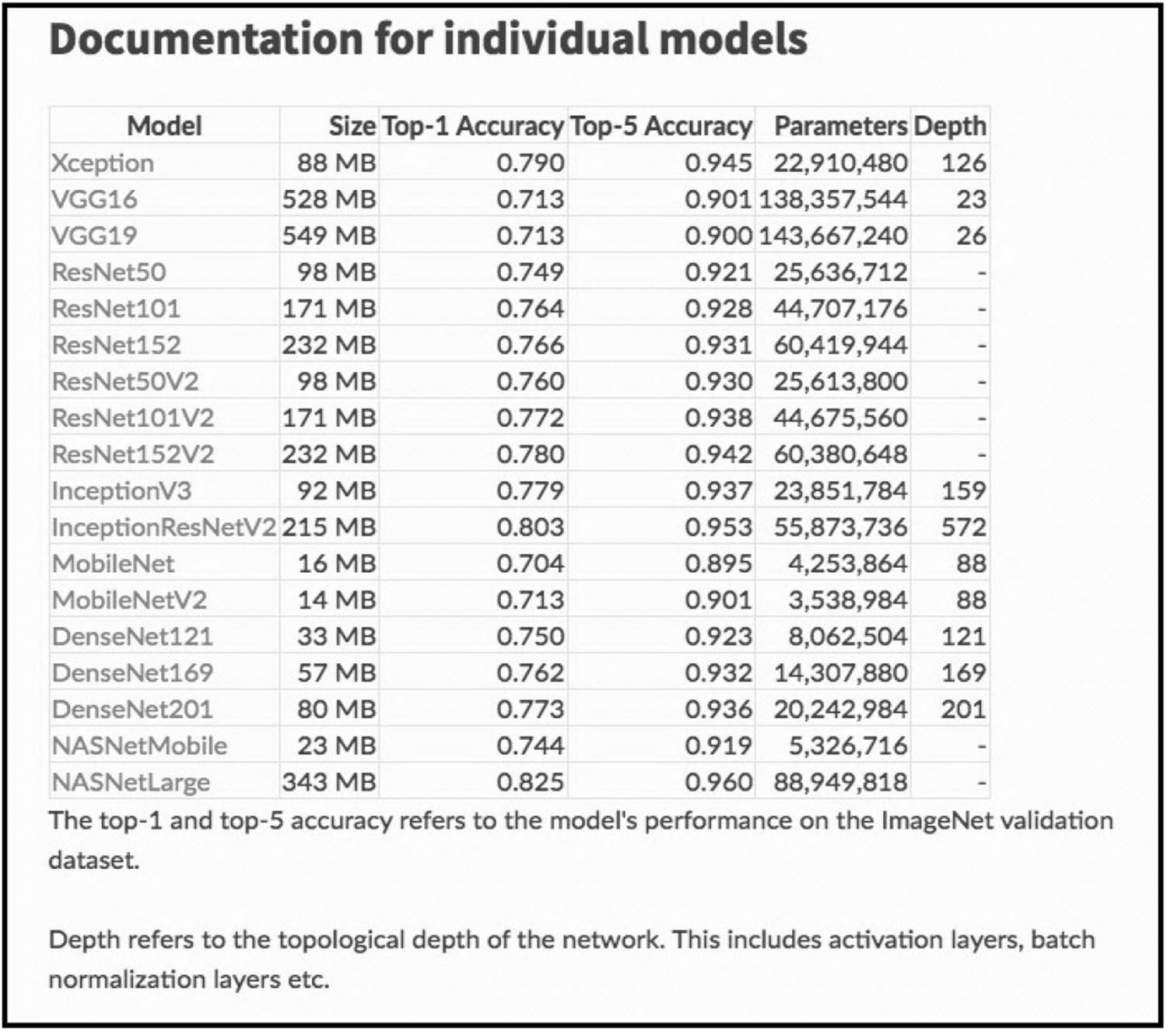


Statistical models have always influenced culture and politics. They did not just emerge with machine learning: machine learning is just a new way to automate the technique of statistical modelling. When Greta Thunberg warns ‘Listen to science.’ what she really means, being a good student of mathematics, is ‘Listen to the statistical models of climate science.’ No statistical models, no climate science: no climate science, no climate activism. Climate science is indeed a good example to start with, in order to understand statistical models. Global warming has been calculated by first collecting a vast dataset of temperatures from Earth’s surface each day of the year, and second, by applying a mathematical model that plots the curve of temperature variations in the past and projects the same pattern into the future (Edwards 2010). Climate models are historical artefacts that are tested and debated within the *scientific community*, and today, also beyond.^21^ Machine learning models, on the contrary, are opaque and inaccessible to community debate. Given the degree of myth-making and social bias around its mathematical constructs, AI has indeed inaugurated the age of *statistical science fiction*. Nooscope is the projector of this large statistical cinema.

## All models are wrong, but some are useful

‘All models are wrong, but some are useful’—the canonical dictum of the British statistician George Box has long encapsulated the logical limitations of statistics and machine learning (Box 1979). This maxim, however, is often used to legitimise the bias of corporate and state AI. Computer scientists argue that human cognition reflects the capacity to abstract and approximate patterns. Therefore, what’s the problem with machines being approximate, and doing the same? Within this argument, it is rhetorically repeated that ‘the map is not the territory’. This sounds reasonable. But what should be contested is that AI is a heavily compressed and distorted map of the territory and that this map, like many forms of automation, is not open to community negotiation. AI is a map of the territory without community access and community consent.^22^

How does machine learning plot a statistical map of the world? Let’s face the specific case of image recognition (the basic form of the *labour of perception*, which has been codified and automated as pattern recognition)^23^ (Beller 2012). Given an image to be classified, the algorithm detects the edges of an object as the statistical distribution of dark pixels surrounded by light ones (a typical visual pattern). The algorithm does not know what an image is, does not perceive an image as human cognition does, it only computes pixels, numerical values of brightness and proximity. The algorithm is programmed to record only the dark edge of a profile (that is to *fit* that desired pattern) and not all the pixels across the image (that would result in *overfitting* and repeating the whole visual field). A statistical model is said to be trained successfully when it can elegantly *fit* only the important patterns of the training data and apply those patterns also to new data ‘in the wild’. If a model learns the training data too well, it recognises only exact matches of the original patterns and will overlook those with close similarities, ‘in the wild’. In this case, the model is *overfitting*, because it has meticulously learnt everything (including noise) and is not able to distinguish a pattern from its background. On the other hand, the model is *underfitting* when it is not able to detect meaningful patterns from the training data. The notions of data overfitting, fitting and underfitting can be visualised on a Cartesian plane.
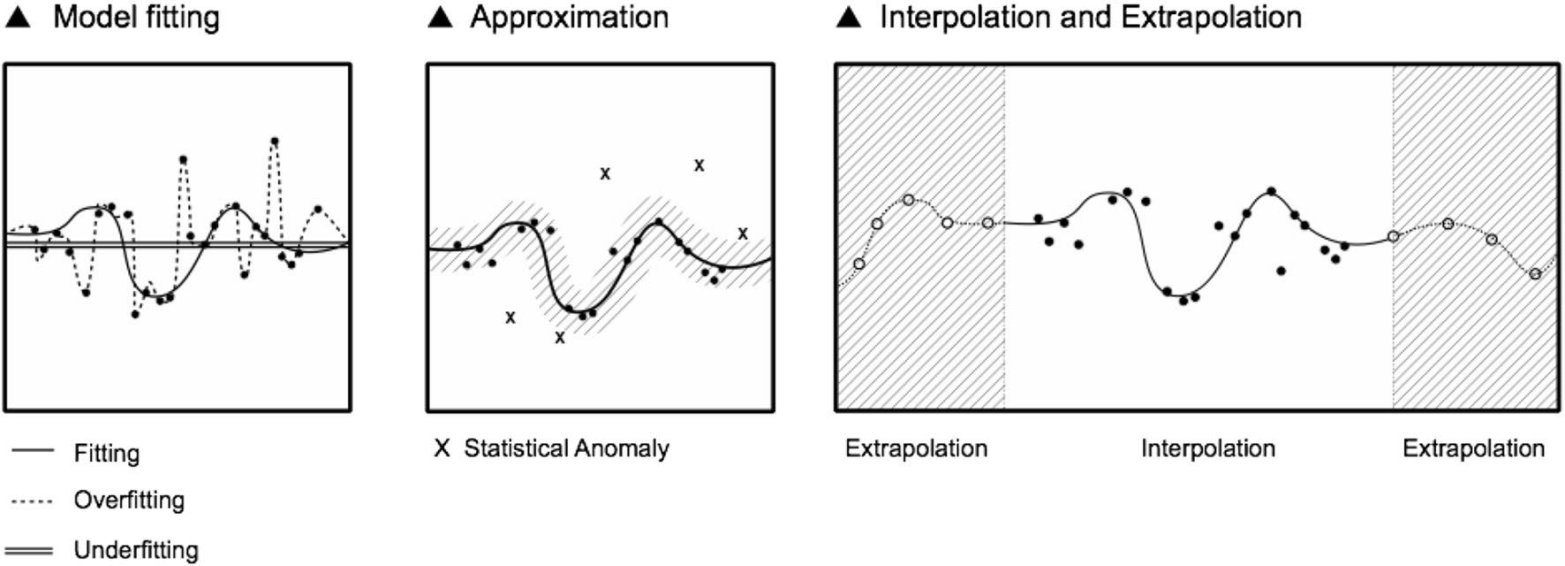


The challenge of guarding the accuracy of machine learning lays in calibrating the equilibrium between data underfitting and overfitting, which is difficult to do because of different machine biases. Machine learning is a term that, as much as ‘AI', anthropomorphizes a piece of technology: machine learning *learns nothing* in the proper sense of the word, as a human does; machine learning simply maps a statistical distribution of numerical values and draws a mathematical function that hopefully approximates human comprehension. That being said, machine learning can, for this reason, cast new light on the ways in which humans comprehend.

The statistical model of machine learning algorithms is also an approximation in the sense that it guesses the missing parts of the data graph: either through *interpolation*, which is the prediction of an output *y* within the known interval of the input *x* in the training dataset, or through *extrapolation,* which is the prediction of output *y* beyond the limits of *x*, often with high risks of inaccuracy. This is what ‘intelligence’ means today within machine intelligence: to extrapolate a non-linear function beyond known data boundaries. As Dan McQuillian aptly puts it: ‘There is no intelligence in artificial intelligence, nor does it learn, even though its technical name is machine learning, it is simply mathematical minimization’ (McQuillan 2018a; b).

It is important to recall that the ‘intelligence’ of machine learning is not driven by exact formulas of mathematical analysis, but by algorithms of *brute force approximation*. The shape of the correlation function between input *x* and output *y* is calculated algorithmically, step by step, through tiresome mechanical processes of gradual adjustment (like gradient descent, for instance) that are equivalent to the differential calculus of Leibniz and Newton. Neural networks are said to be among the most efficient algorithms, because these differential methods can *approximate* the shape of any function given enough layers of neurons and abundant computing resources.^24^ Brute-force gradual approximation of a function is the core feature of today’s AI, and only from this perspective can one understand its potentialities and limitations—particularly, its escalating carbon footprint (the training of deep neural networks requires exorbitant amounts of energy because of gradient descent and similar training algorithms that operate on the basis of continuous infinitesimal adjustments) (Ganesh et al. 2019).
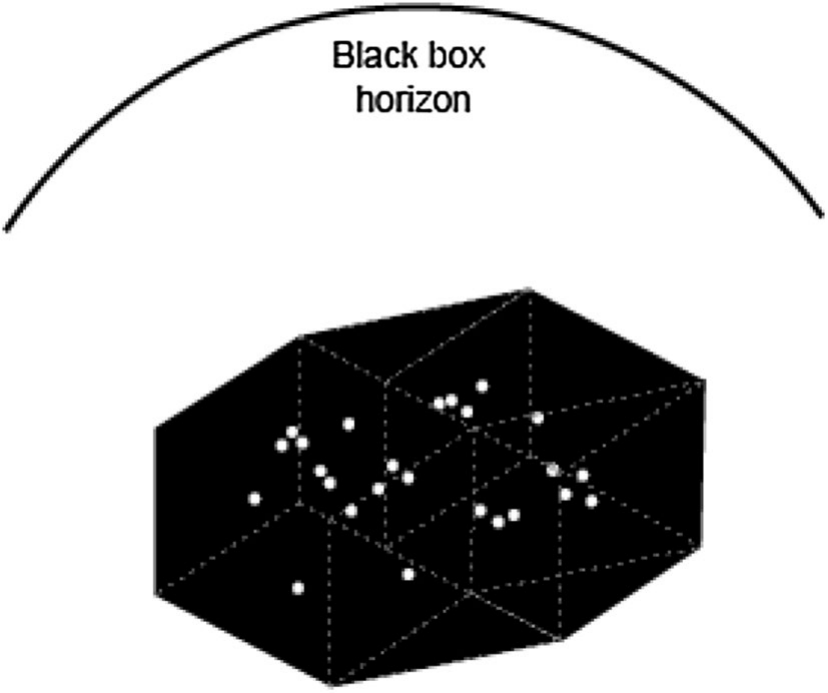


Multidimensional vector space.

## World to vector

The notions of data fitting, overfitting, underfitting, interpolation and extrapolation can be easily visualised in two dimensions, but statistical models usually operate along multidimensional spaces of data. Before being analysed, data are encoded into a *multi-dimensional vector space* that is far from intuitive. What is a vector space and why is it multi-dimensional? Cardon, Cointet and Mazière describe the vectorialisation of data in this way:

A neural network requires the inputs of the calculator to take on the form of a vector. Therefore, the world must be coded in advance in the form of a purely digital vectorial representation. While certain objects such as images are naturally broken down into vectors, other objects need to be ‘embedded’ within a vectorial space before it is possible to calculate or classify them with neural networks. This is the case of text, which is the prototypical example. To input a word into a neural network, the *Word2vec* technique ‘embeds’ it into a vectorial space that measures its distance from the other words in the corpus. Words thus inherit a position within a space with several hundreds of dimensions. The advantage of such a representation resides in the numerous operations offered by such a transformation. Two terms whose inferred positions are near one another in this space are equally similar semantically; these representations are said to be distributed: the vector of the concept ‘apartment’ [− 0.2, 0.3, − 4.2, 5.1…] will be similar to that of ‘house’ [− 0.2, 0.3, − 4.0, 5.1…].[…] While natural language processing was pioneering for ‘embedding’ words in a vectorial space, today we are witnessing a generalization of the embedding process which is progressively extending to all applications fields: networks are becoming simple points in a vectorial space with *graph2vec*, texts with *paragraph2vec*, films with *movie2vec*, meanings of words with *sens2vec*, molecular structures with *mol2vec*, etc. According to Yann LeCun, the goal of the designers of connectionist machines is to put the world in a vector (*world2vec*) (Cardon et al. 2018a).

*Multi-dimensional vector space* is another reason why the logic of machine learning is difficult to grasp. Vector space is another new cultural technique, worth becoming familiar with. The field of Digital Humanities, in particular, has been covering the technique of vectorialisation through which our collective knowledge is invisibly rendered and processed. William Gibson’s original definition of cyberspace prophesized, most likely, the coming of a vector space rather than virtual reality: ‘A graphic representation of data abstracted from the banks of every computer in the human system. Unthinkable complexity. Lines of light ranged in the nonspace of the mind, clusters and constellations of data. Like city lights, receding’ (Gibson 1984, p. 69).
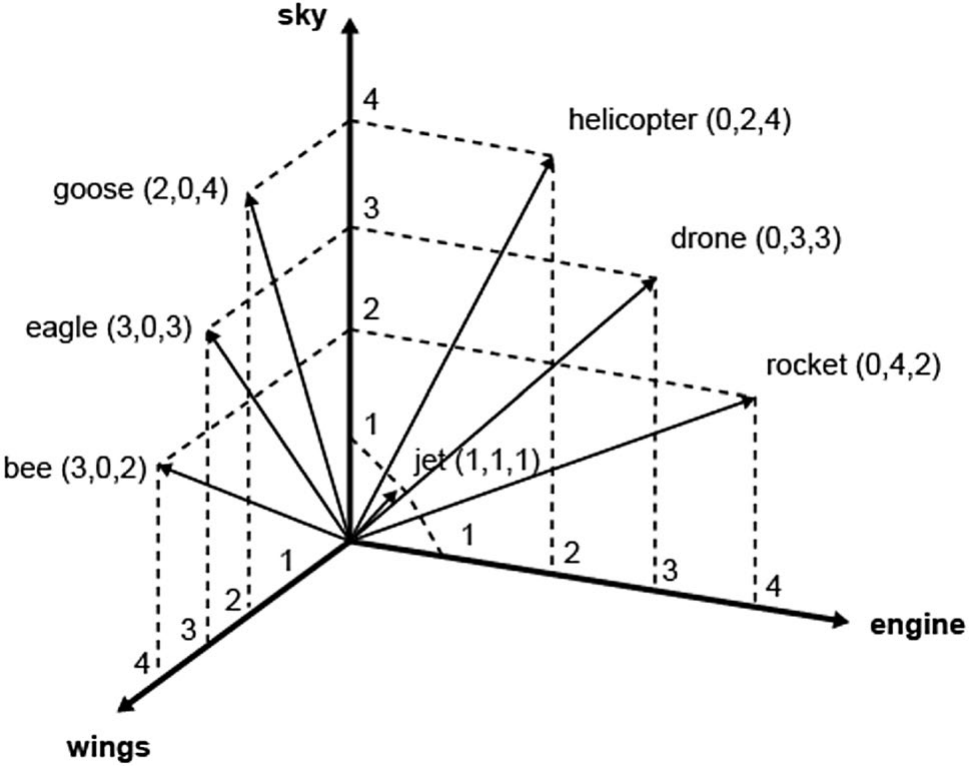


Vector space of seven words in three contexts.^25^

It must be stressed, however, that machine learning still resembles more craftsmanship than exact mathematics. AI is still a history of hacks and tricks rather than mystical intuitions. For example, one trick of information compression is *dimensionality reduction*, which is used to avoid the Curse of Dimensionality, that is the exponential growth of the variety of features in the vector space. The dimensions of the categories that show low variance in the vector space (i.e. whose values fluctuate only a little) are aggregated to reduce calculation costs. Dimensionality reduction can be used to cluster word meanings (such as in the model word2vec) but can also lead to *category reduction*, which can have an impact on the representation of social diversity. Dimensionality reduction can shrink taxonomies and introduce bias, further normalising world diversity and obliterating unique identities (Samadi et al. 2018).
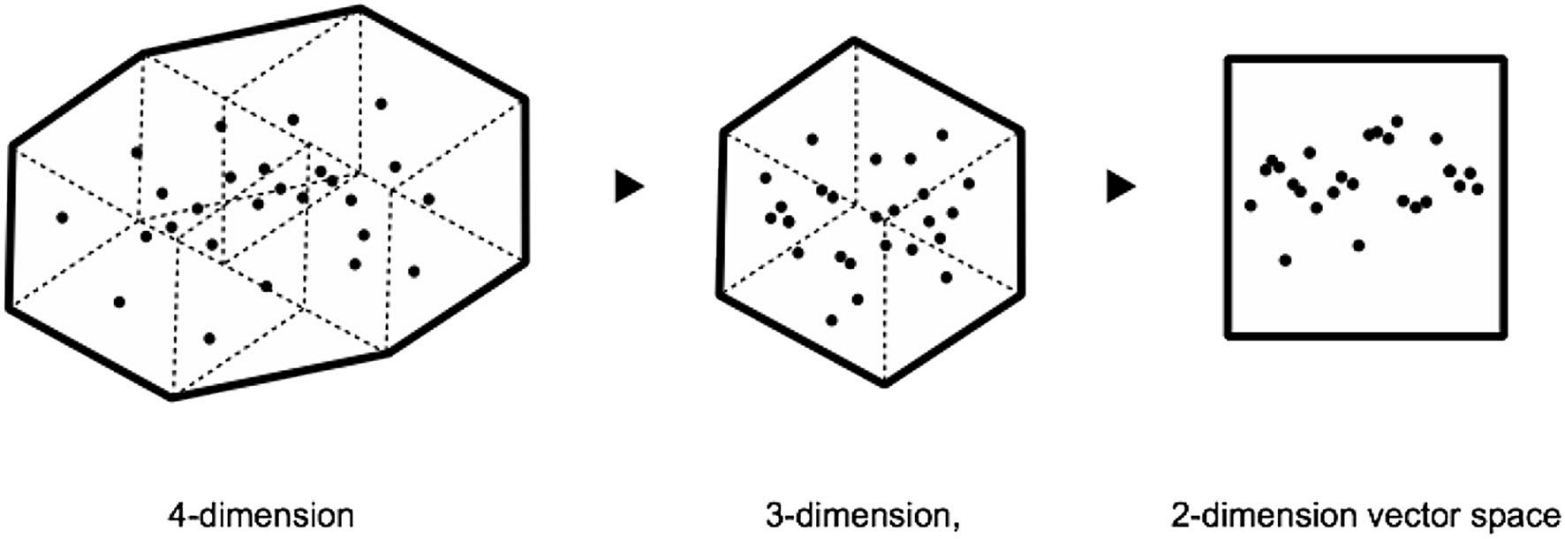


## The society of classification and prediction bots

Most of the contemporary applications of machine learning can be described according to the two modalities of classification and prediction, which outline the contours of a new society of control and statistical governance. Classification is known as *pattern recognition,* while prediction can be defined also as *pattern generation*. A new pattern is recognised or generated by interrogating the inner core of the statistical model.

Machine learning *classification* is usually employed to recognise a sign, an object, or a human face, and to assign a corresponding category (label) according to taxonomy or cultural convention. An input file (e.g. a headshot captured by a surveillance camera) is run through the model to determine whether it falls within its statistical distribution or not. If so, it is assigned the corresponding output label. Since the times of the Perceptron, classification has been the originary application of neural networks: with Deep Learning, this technique is found ubiquitously in face recognition classifiers that are deployed by police forces and smartphone manufacturers alike.

Machine learning *prediction* is used to project future trends and behaviours according to past ones, that is to complete a piece of information knowing only a portion of it. In the prediction modality, a small sample of input data (a primer) is used to predict the missing part of the information following once again the statistical distribution of the model (this could be the part of a numerical graph oriented toward the future or the missing part of an image or audio file). Incidentally, other modalities of machine learning exist: the statistical distribution of a model can be dynamically visualised through a technique called latent space exploration and, in some recent design applications, also *pattern exploration.*^26^

Machine learning classification and prediction are becoming ubiquitous techniques that constitute new forms of surveillance and governance. Some apparatuses, such as self-driving vehicles and industrial robots, can be an integration of both modalities. A self-driving vehicle is trained to recognise different objects on the road (people, cars, obstacles, signs) and predict future actions based on decisions that a human driver has taken in similar circumstances. Even if recognising an obstacle on a road seems to be a neutral gesture (it’s not), identifying a human being according to categories of gender, race and class (and in the recent COVID-19 pandemic as sick or immune), as state institutions are increasingly doing, is the gesture of a new disciplinary regime. The hubris of automated classification has caused the revival of reactionary Lombrosian techniques that were thought to have been consigned to history, techniques such as automatic gender recognition (AGR), ‘a subfield of facial recognition that aims to algorithmically identify the gender of individuals from photographs or videos’ (Keyes 2018).

Recently, the generative modality of machine learning has had a cultural impact: its use in the production of visual artefacts has been received by mass media as the idea that artificial intelligence is ‘creative’ and can autonomously make art. An artwork that is said to be created by AI always hides a human operator, who has applied the generative modality of a neural network trained on a specific dataset. In this modality, the neural network is run *backwards* (moving from the smaller output layer toward the larger input layer) to generate new patterns after being trained at classifying them, a process that usually moves from the larger input layer to the smaller output layer. The generative modality, however, has some useful applications; it can be used as a sort of reality check to reveal what the model has learnt, i.e. to show how the model ‘sees the world.’ It can be applied to the model of a self-driving car, for instance, to check how the road scenario is projected.

A famous way to illustrate how a statistical model ‘sees the world’ is Google DeepDream. DeepDream is a convolutional neural network based on Inception (which is trained on the ImageNet dataset mentioned above) that was programmed by Alexander Mordvintsev to project hallucinatory patterns. Mordvintsev had the idea to ‘turn the network upside down’, that is to turn a classifier into a generator, using some random noise or generic landscape images as input (Mordvintsev et al. 2015). He discovered that ‘neural networks that were trained to discriminate between different kinds of images have quite a bit of the information needed to generate images too.’ In DeepDream first experiments, bird feathers and dog eyes started to emerge everywhere as dog breeds and bird species are vastly overrepresented in ImageNet. It was also discovered that the category ‘dumbbell’ was learnt with a surreal human arm always attached to it. Proof that many other categories of ImageNet are misrepresented.

The two main modalities of classification and generation can be assembled in further architectures such as in the Generative Adversarial Networks. In the GAN architecture, a neural network with the role of *discriminator* (a traditional classifier) has to recognise an image produced by a neural network with the role of *generator,* in a reinforcement loop that trains the two statistical models simultaneously. For some converging properties of their respective statistical models, GANs have proved very good at generating highly realistic pictures. This ability has prompted their abuse in the fabrication of ‘deep fakes’.^27^ Concerning regimes of truth, a similar controversial application is the use of GANs to generate synthetic data in cancer research, in which neural networks trained on unbalanced datasets of cancer tissues have started to hallucinate cancer where there was none (Cohen et al. 2018). In this case ‘instead of discovering things, we are inventing things', Fabian Offert notices, ‘the space of discovery is identical to the space of knowledge that the GAN has already had.[…] While we think that we are seeing through GAN—looking at something with the help of a GAN—we are actually seeing *into* a GAN. GAN vision is not augmented reality, it is virtual reality. GANs do blur discovery and invention’ (Offert 2020). The GAN simulation of brain cancer is a tragic example of AI-driven scientific hallucination.
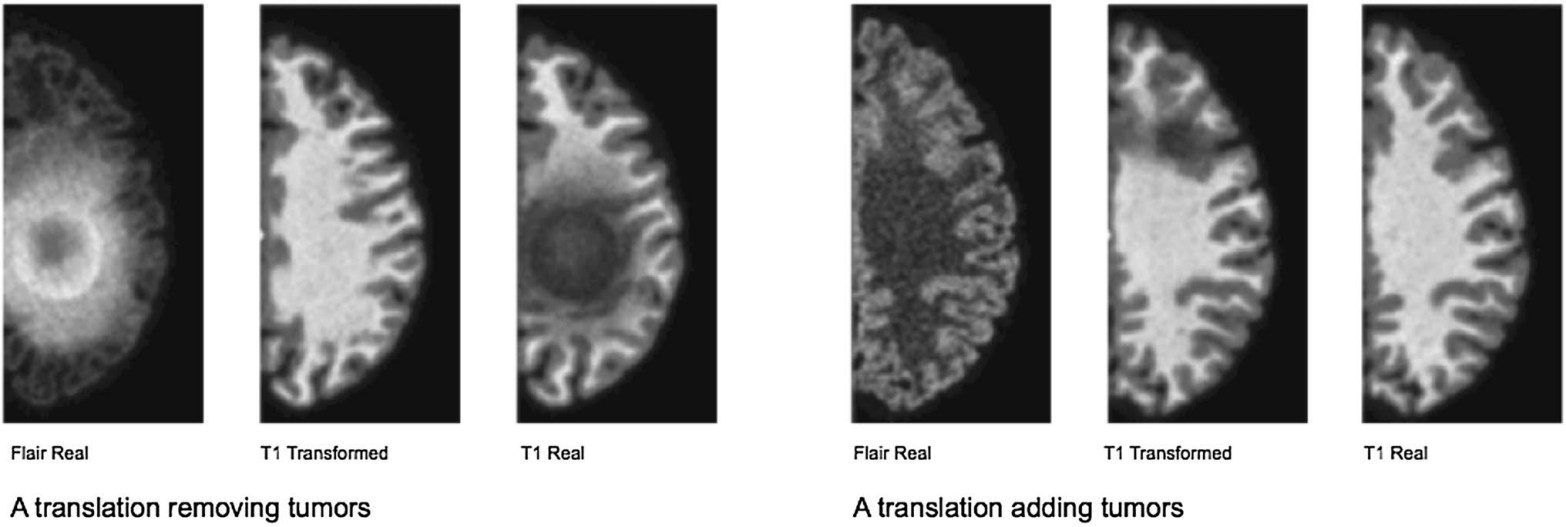


Joseph Paul Cohen, Margaux Luck and Sina Honari. ‘Distribution Matching Losses Can Hallucinate Features in Medical Image Translation’, 2018. Courtesy of the authors.

## Faults of a statistical instrument: the undetection of the new

The normative power of AI in the twenty first century has to be scrutinised in these epistemic terms: what does it mean to frame collective knowledge as patterns, and what does it mean to draw vector spaces and statistical distributions of social behaviours? According to Foucault, in early modern France, statistical power was already used to measure social norms, discriminating between normal and abnormal behaviour (Foucault 2004, p. 26). AI easily extends the ‘power of normalisation’ of modern institutions, among others bureaucracy, medicine and statistics (originally, the numerical knowledge possessed by the state about its population) that passes now into the hands of AI corporations. The institutional norm has become a computational one: the classification of the subject, of bodies and behaviours, seems no longer to be an affair for public registers, but instead for algorithms and datacentres.^28^ ‘Data-centric rationality’, Paula Duarte has concluded, ‘should be understood as an expression of the coloniality of power’ (Ricaurte 2019).

A gap, a friction, a conflict, however, always persists between AI statistical models and the human subject that is supposed to be measured and controlled. This logical gap between AI statistical models and society is usually debated as *bias*. It has been extensively demonstrated how face recognition misrepresents social minorities and how black neighbourhoods, for instance, are bypassed by AI-driven logistics and delivery service (Ingold and Soper 2016). If gender, race and class discriminations are amplified by AI algorithms, this is also part of a larger problem of discrimination and normalisation at the logical core of machine learning. The logical and political limitation of AI is the technology’s difficulty in the *recognition and prediction of a new event*. How is machine learning dealing with a truly unique anomaly, an uncommon social behaviour, an innovative act of disruption? The two modalities of machine learning display a limitation that is not simply bias.

A logical limit of machine learning classification, or pattern recognition, is the inability to recognise a *unique anomaly* that appears for the first time, such as a new metaphor in poetry, a new joke in everyday conversation, or an unusual obstacle (a pedestrian? a plastic bag?) on the road scenario. The *undetection of the new* (something that has never ‘been seen’ by a model and therefore never classified before in a known category) is a particularly hazardous problem for self-driving cars and one that has already caused fatalities. Machine learning prediction, or pattern generation, show similar faults in the guessing of future trends and behaviours. As a technique of information compression, machine learning automates the dictatorship of the past, of past taxonomies and behavioural patterns, over the present. This problem can be termed the *regeneration of the old*—the application of a homogenous space–time view that restrains the possibility of a new historical event.

Interestingly, in machine learning, the logical definition of a security issue also describes the logical limit of its creative potential. The problems characteristic of the *prediction of the new* are logically related to those that characterise the *generation of the new*, because the way a machine learning algorithm predicts a trend on a time chart is identical to the way it generates a new artwork from learnt patterns. The hackneyed question ‘Can AI be creative?’ should be reformulated in technical terms: is machine learning able to create works that are not imitations of the past? Is machine learning able to extrapolate beyond the stylistic boundaries of its training data? The ‘creativity’ of machine learning is limited to the detection of styles from the training data and then random improvisation within these styles. In other words, machine learning can explore and improvise only within the logical boundaries that are set by the training data. For all these issues, and its degree of information compression, it would be more accurate to term machine learning art as *statistical art.*
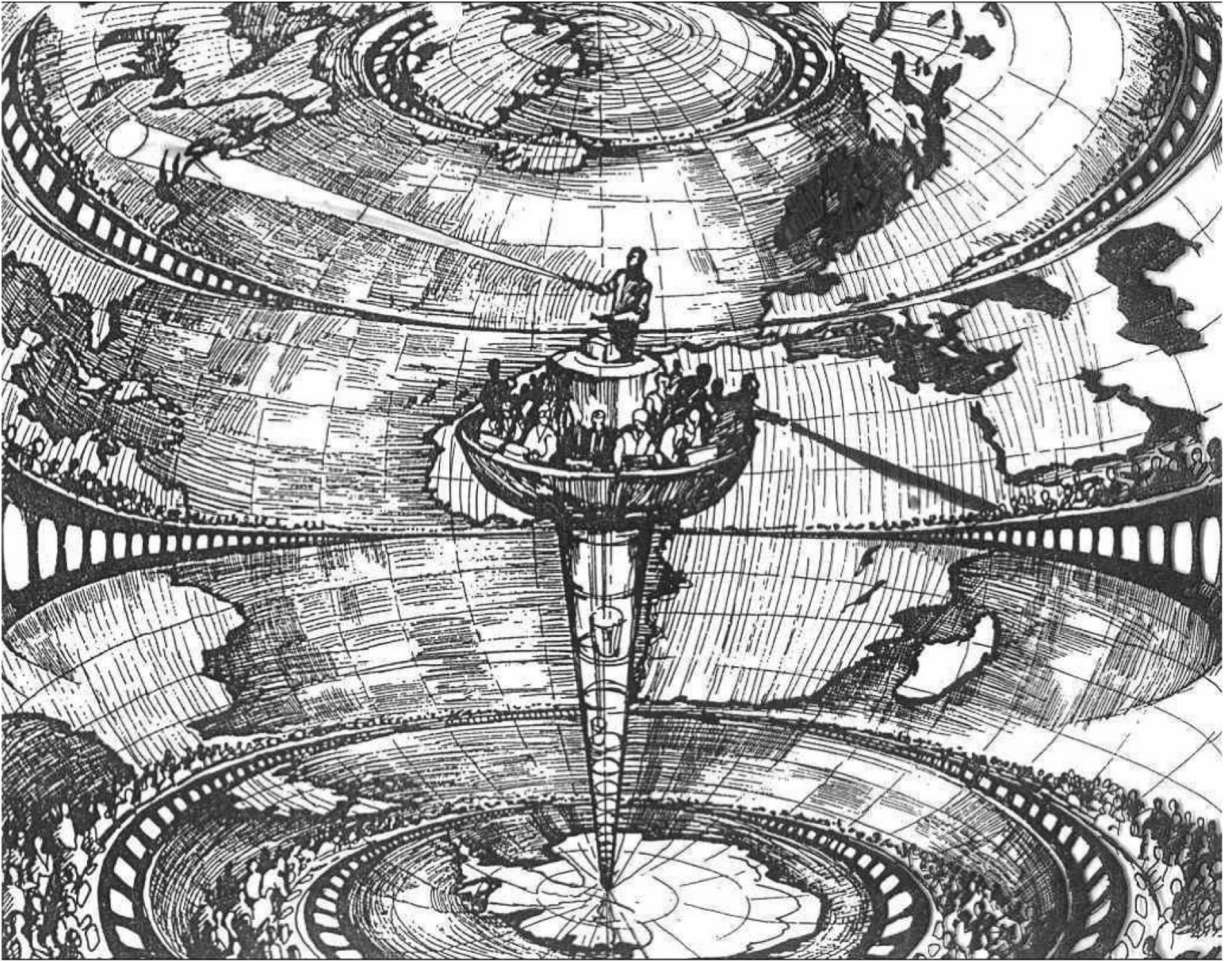


Lewis Fry Richardson, *Weather Prediction by Numerical Process*, Cambridge University Press, 1922.

Another unspoken bug of machine learning is that the statistical correlation between two phenomena is often adopted to explain causation from one to the other. In statistics, it is commonly understood that *correlation does not imply causation*, meaning that a statistical coincidence alone is not sufficient to demonstrate causation. A tragic example can be found in the work of statistician Frederick Hoffman, who in 1896 published a 330-page report for insurance companies to demonstrate a *racial correlation* between being a black American and having short life expectancy (O’Neil 2016). Superficially mining data, machine learning can construct any arbitrary correlation that is then perceived as real. In 2008, this logical fallacy was proudly embraced by Wired director Chris Anderson who declared the ‘end of theory’, because ‘the data deluge makes the scientific method obsolete’ (Anderson 2008).^29^ According to Anderson, himself no expert on scientific method and logical inference, statistical correlation is enough for Google to run its ads business, therefore, it must also be good enough to automatically discover scientific paradigms. Even Judea Pearl, a pioneer of Bayesian networks, believes that machine learning is obsessed with ‘curve fitting’, recording correlations without providing explanations (Mackenzie and Judea 2018). Such a logical fallacy has already become a political one, if one considers that police forces worldwide have adopted predictive policing algorithms.^30^ According to Dan McQuillan, when machine learning is applied to society in this way, it turns into a biopolitical apparatus of *preemption*, that produces subjectivities which can subsequently be criminalized (McQuillan 2018a; b). Ultimately, machine learning obsessed with ‘curve fitting’ imposes a *statistical culture* and replaces the traditional episteme of causation (and political accountability) with one of correlations blindly driven by the automation of decision making.

## Adversarial intelligence vs. artificial intelligence

So far, the statistical diffractions and hallucinations of machine learning have been followed step by step through the multiple lenses of the Nooscope. At this point, the orientation of the instrument has to be reversed: scientific theories as much as computational devices are inclined to consolidate an abstract perspective—the scientific ‘view from nowhere’, that is often just the point of view of power. The obsessive study of AI can suck the scholar into an abyss of computation and the illusion that the technical form illuminates the social one. As Paola Ricaurte remarks: ‘Data extractivism assumes that everything is a data source’ (Ricaurte 2019). How to emancipate ourselves from a data-centric view of the world? It is time to realise that it is not the statistical model that constructs the subject, but rather the subject that structures the statistical model. Internalist and externalist studies of AI have to blur: subjectivities make the mathematics of control from within, not from without. To second what Guattari once said of machines in general, machine intelligence too is constituted of ‘hyper-developed and hyper-concentrated forms of certain aspects of human subjectivity’ (Guattari 2013, p. 2).

Rather than studying only how technology works, critical inquiry studies also how it breaks, how subjects rebel against its normative control and workers sabotage its gears. In this sense, a way to sound the limits of AI is to look at hacking practices. Hacking is an important method of knowledge production, a crucial epistemic probe into the obscurity of AI.^31^ Deep learning systems for face recognition have triggered, for instance, forms of counter-surveillance activism. Through techniques of face obfuscation, humans have decided to become unintelligible to artificial intelligence: that is to become, themselves, *black boxes*. The traditional techniques of *obfuscation* against surveillance immediately acquire a mathematical dimension in the age of machine learning. For example, AI artist and researcher Adam Harvey has invented a camouflage textile called HyperFace that fools computer vision algorithms to see multiple human faces where there is none (Harvey 2016). Harvey's work provokes the question: what constitutes a face for a human eye, on the one hand, and a computer vision algorithm, on the other? The neural glitches of HyperFace exploit such a cognitive gap and reveal what a human face looks like to a machine. This gap between human and machine perception helps to introduce the growing field of adversarial attacks.
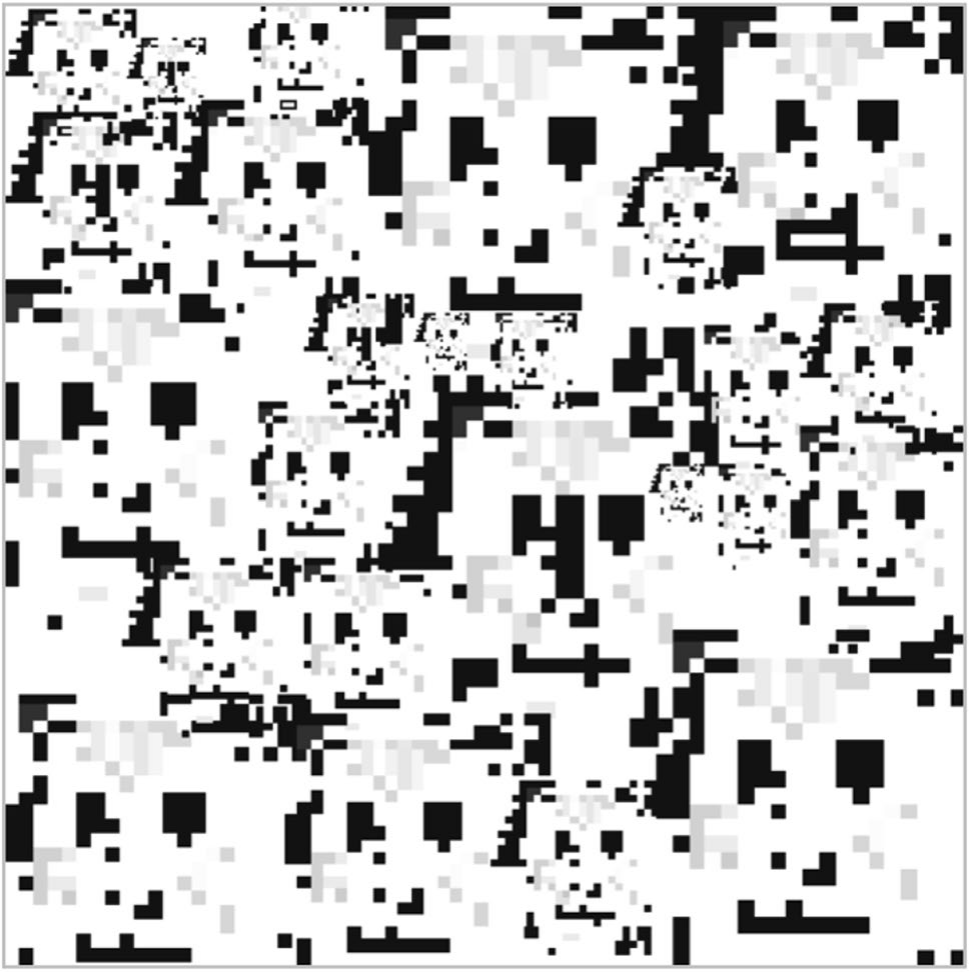


Adam Harvey, HyperFace pattern, 2016.

*Adversarial attacks* exploit blind spots and weak regions in the statistical model of a neural network, usually to fool a classifier and make it perceive something that is not there. In object recognition, an adversarial example can be a doctored image of a turtle, which looks innocuous to a human eye but gets misclassified by a neural network as a rifle (Athalye et al. 2017). Adversarial examples can be realised as 3D objects and even stickers for road signs that can misguide self-driving cars (which may read a speed limit of 120 km/h where it is actually 50 km/h) (Morgulis et al. 2019). Adversarial examples are designed knowing what a machine has never seen before. This effect is achieved also by reverse-engineering the statistical model or by polluting the training dataset. In this latter sense, the technique of *data poisoning* targets the training dataset and introduces doctored data. In doing so, it alters the accuracy of the statistical model and creates a backdoor that can be eventually exploited by an adversarial attack.^32^

Adversarial attack seems to point to a mathematical vulnerability that is common to all machine learning models: ‘An intriguing aspect of adversarial examples is that an example generated for one model is often misclassified by other models, even when they have different architectures or were trained on disjoint training sets’ (Goodfellow et al. 2014). Adversarial attacks remind us of the discrepancy between human and machine perception and that the logical limit of machine learning is also a political one. The logical and ontological boundary of machine learning is the unruly subject or anomalous event that escapes classification and control. The subject of algorithmic control fires back. Adversarial attacks are a way to sabotage the assembly line of machine learning by inventing a virtual obstacle that can set the control apparatus out of joint. An adversarial example is the *sabot* in the age of AI.

## Labour in the age of AI

The natures of the ‘input’ and ‘output’ of machine learning have to be clarified. AI troubles are not only about information bias but also labour. AI is not just a control apparatus, but also a productive one. As just mentioned, an invisible workforce is involved in each step of its assembly line (dataset composition, algorithm supervision, model evaluation, etc.). Pipelines of endless tasks innervate from the Global North into the Global South; crowdsourced platforms of workers from Venezuela, Brazil and Italy, for instance, are crucial to teach German self-driving cars ‘how to see’ (Schmidt 2019). Against the idea of alien intelligence at work, it must be stressed that in the whole computing process of AI the human worker has never left the loop, or put more accurately, has never left the assembly line. Mary Gray and Siddharth Suri coined the term ‘ghost work’ for the invisible labour that makes AI appear artificially autonomous.

Beyond some basic decisions, today’s artificial intelligence can’t function without humans in the loop. Whether it’s delivering a relevant newsfeed or carrying out a complicated texted-in pizza order, when the artificial intelligence (AI) trips up or can’t finish the job, thousands of businesses call on people to quietly complete the project. This new digital assembly line aggregates the collective input of distributed workers, ships pieces of projects rather than products, and operates across a host of economic sectors at all times of the day and night.

Automation is a myth, because machines, including AI, constantly call for human help, some authors have suggested replacing ‘automation’ with the more accurate term *heteromation* (Ekbia and Nardi 2017). Heteromation means that the familiar narrative of AI as *perpetuum mobile* is possible only thanks to a reserve army of workers.

Yet, there is a more profound way in which labour constitutes AI. The information source of machine learning (whatever its name: input data, training data or just data) is always a representation of human skills, activities and behaviours, social production at large. All training datasets are, implicitly, a diagram of the division of human labour that AI has to analyse and automate. Datasets for image recognition, for instance, record the visual labour that drivers, guards, and supervisors usually perform during their tasks. Even scientific datasets rely on scientific labour, experiment planning, laboratory organisation, and analytical observation. The information flow of AI has to be understood as an apparatus designed to extract ‘analytical intelligence’ from the most diverse forms of labour and to transfer such intelligence into a machine (obviously including, within the definition of labour, extended forms of social, cultural and scientific production).^33^ In short, the origin of machine intelligence is the *division of labour* and its main purpose is the *automation of labour*.

Historians of computation have already stressed the early steps of machine intelligence in the nineteenth century project of mechanizing the division of mental labour, specifically the task of hand calculation (Schaffer 1994; Daston 1994; Jones 2016). The enterprise of computation has since then been a combination of surveillance and disciplining of labour, of optimal calculation of surplus-value, and planning of collective behaviours (Pasquinelli 2019a). Computation was established by and still enforces a regime of visibility and intelligibility, not just of logical reasoning. The genealogy of AI as an apparatus of power is confirmed today by its widespread employment in technologies of identification and prediction, yet the core anomaly which always remains to be computed is the *disorganisation of labour*.

As a technology of automation, AI will have a tremendous impact on the job market. If Deep Learning has a 1% error rate in image recognition, for example, it means that roughly 99% of routine work based on visual tasks (e.g. airport security) can be potentially replaced (legal restrictions and trade union opposition permitting). The impact of AI on labour is well described (from the perspective of workers, finally) within a paper from the European Trade Union Institute, which highlights ‘seven essential dimensions that future regulation should address to protect workers: (1) safeguarding worker privacy and data protection; (2) addressing surveillance, tracking and monitoring; (3) making the purpose of AI algorithms transparent; (4) ensuring the exercise of the ‘right to explanation’ regarding decisions made by algorithms or machine learning models; (5) preserving the security and safety of workers in human–machine interactions; (6) boosting workers’ autonomy in human–machine interactions; (7) enabling workers to become AI literate’ (Ponce 2020).

Ultimately, the Nooscope manifests in response to the need for a novel Machinery Question in the age of AI. The Machinery Question was a debate that sparked in England during the industrial revolution, when the response to the employment of machines and workers’ unemployment was a social campaign for more education about machines, that took the form of the Mechanics’ Institute Movement (Berg 1980).^34^ Today, an Intelligent Machinery Question is needed to develop more collective intelligence about machine intelligence, more public education instead of ‘learning machines’ and their regime of knowledge extractivism, which crosses once again old colonial routes (if one looks at the network map of crowdsourcing). Also in the Global North, the colonial relationship between corporate AI and the production of knowledge as a common good has to be brought to the forefront. The Nooscope’s purpose is to break into the hidden room of the corporate Mechanical Turk, and to illuminate the invisible labour of knowledge that makes machine intelligence appear ideologically alive.

*Thanks to Jon Beller, Claire Glanois, Adam Harvey, Leonardo Impett, Arif Kornweitz, Wietske Maas, Dan McQuillan, Fabian Offert, Godofredo Pereira, Mitch Speed and the extended community around KIM HfG Karlsruhe for their inputs and comments.*

## Footnotes

1 On the autonomy of technology see: Winner (2001).

2 For the colonial extensions of the operations of logistics, algorithms and finance see: Mezzadra and Neilson (2019). On the epistemic colonialism of AI see: Pasquinelli (2019b).

3 Digital humanities term a similar technique *distant reading*, which has gradually involved data analytics and machine learning in literary and art history. See: Moretti (2013).

4 For a concise history of AI see: Cardon et al. (2018b).

5 The use of the visual analogy is also intended to record the fading distinction between image and logic, representation and inference, in the technical composition of AI. The statistical models of machine learning are operative representations (in the sense of Harun Farocki’s operative images).

6 For a systematic study of the logical limitations of machine learning see: Malik (2002).

7 For a more detailed list of AI biases see: Guttag and Suresh (2019) and Galstyan et al. (2019).

8 See also: Crawford (2017).

9 Computer scientists argue that AI belongs to a subfield of *signal processing*, that is *data compression.*

10 Projects such as Explainable Artificial Intelligence, Interpretable Deep Learning and Heatmapping among others have demonstrated that breaking into the ‘black box’ of machine learning is possible. Nevertheless, the full interpretability and explicability of machine learning statistical models remains a myth. See: Lipton (2016).

11 In supervised learning. Also self-supervised learning maintains forms of human intervention.

12 On taxonomy as a form of knowledge and power see: Foucault (2005).

13 Such as Amazon Mechanical Turk, cynically termed ‘artificial artificial intelligence’ by Jeff Bezos. See: Pontin (2007).

14 Although the convolutional architecture dates back to Yann LeCun’s work in the late 1980s, Deep Learning starts with this paper: Krizhevsky et al. (2017).

15 For an accessible (yet not very critical) account of the ImageNet development see: Mitchell (2019).

16 WordNet is ‘a lexical database of semantic relations between words’ which was initiated by George Armitage at Princeton University in 1985. It provides a strict tree-like structure of definitions.

17 The GDPR data privacy regulation that was passed by the European Parliament in May 2018 is, however, an improvement compared to the regulation that is missing in the United States.

18 The parameters of a model that are learnt from data are called ‘parameters’, while parameters that are not learnt from data and are fixed manually are called ‘hyperparameters’ (these determine number and properties of the parameters.).

19 This value can be also a percentage value between 1 and 0.

20 https://keras.io/applications (documentation for individual models.)

21 See the Community Earth System Model (CESM) that has been developed by the National Center for Atmospheric Research in Bolder, Colorado, since 1996. The Community Earth System Model is a fully coupled numerical simulation of the Earth system consisting of atmospheric, ocean, ice, land surface, carbon cycle, and other components. CESM includes a climate model providing state-of-the-art simulations of the Earth’s past, present, and future.’ https://www.cesm.ucar.edu

22 Post-colonial and post-structuralist schools of anthropology and ethnology have stressed that there is never territory per se, but always an act of territorialisation.

23 Pattern recognition is one among many other economies of attention. ‘To look is to labor’, as Jonathan Beller reminds us.

24 As proven by the Universal Approximation Theorem.

25 Source: https://corpling.hypotheses.org/495.

26 See the idea of assisted and generative creation in: Pieters and Winiger (2016).

27 Deep fakes are synthetic media like videos in which a person's face is replaced with someone else’s facial features, often for the purpose to forge fake news.

28 On computational norms see: Pasquinelli (2017).

29 For a critique see: Mazzocchi (2015).

30 Experiments by the New York Police Department since the late 1980s. See: Pasquinelli, Arcana Mathematica Imperii.

31 The relationship between AI and hacking is not as antagonistic as it may appear: it often resolves in a loop of mutual learning, evaluation and reinforcement.

32 Data poisoning can also be employed to protect privacy by entering anonymised or random information into the dataset.

33 For the idea of analytical intelligence see: Daston (2018).

34 In fact, even the Economist has recently warned about ‘the return of the machinery question’ in the age of AI. See: Standage (2016).
